# Visual search performance depends on the congruency of olfactory sensations

**DOI:** 10.1038/s41598-025-25995-1

**Published:** 2025-10-31

**Authors:** Serena Castellotti, Marija Soldo, Tina Plank, Maria Michela Del Viva, Mark W. Greenlee

**Affiliations:** 1 Department of Neurosciences, Psychology, Drug Research and Child Health (NEUROFARBA), University of Florence, Florence, Italy; 2 Department of Translational Research on New Technologies in Medicine and Surgery, University of Pisa, Pisa, Italy; 3 Institute of Psychology, University of Regensburg, Regensburg, Germany

**Keywords:** Visual search, Olfaction, Multisensory integration, Food choices., Psychology, Human behaviour, Sensory processing

## Abstract

We here provide evidence for a benefit of congruent olfactory sensations during a challenging visual search task. Using a four-channel olfactometer, we exposed our participants to one of three suprathreshold fruit odorants (lemon, apple, or strawberry) or neutral room air (no odorant) while they searched for an image of a cued-target fruit presented among fruit distractors. Congruent odorants (e.g., exposure to a lemon scent while searching for a lemon among other fruits) led to greater performance levels and faster responses compared to trials where the participants were exposed to an odorant that was incongruent to the searched-for target fruit or to neutral room air, respectively. Post-hoc correlations across our *n* = 22 participants suggest that low performers in the baseline (no-odorant) search task benefited most from the congruent odorant-visual object coupling, whereas the visual search of high performers was impaired most by incongruent couplings. A control task points to large individual differences in olfactory discrimination across our healthy participants and their discriminative ability correlates with the amplitude and sign of the congruency effect. Our findings point to a multisensory congruency effect of odorant processing while participants visually search for a nutritional item among other food choices, suggesting that your nose knows what you are looking for.

**Supplementary Information:**

The online version contains supplementary material available at 10.1038/s41598-025-25995-1.

## Introduction

Imagine yourself being in a bustling marketplace in search of your favorite fruit. Your mental image of the color and shape of this fruit may help guide your visual search. Could the concurrent sensory processing of the characteristic aroma of this fruit also assist you in finding your choice among the multi-colored fruits that serve to divert your attention?

Visual search of a target among a set of distractors has become a leading paradigm in the study of early visual processing, as this paradigm lends itself to the study of higher-order cognitive processes like attention and working memory^1–5^. Differences in key visual features like color^6^, contrast and orientation^7^, direction and speed of motion^8^, and stereoscopic depth^9,10^ can render a target to be more salient among a myriad of distracting non-targets. Prior information about the possible location of distractors randomly placed with respect to the target can render the target more salient thereby enhancing search performance^11,12^. Performance and search times are important indicators of the underlying perceptual and cognitive processes that render a target more salient when presented among distractors^13^. Multisensory cues can provide a benefit in visual search tasks. A semantically congruent sound can enhance performance in a forced-choice spatial audiovisual congruency task^14^. Even in cases where the multisensory cue (e.g., an auditory tone) is spatially uninformative and task irrelevant^15^, its presence can benefit visual search.

In humans, olfaction plays a critical role in appetitive behavior in choosing between edible foods and palatable beverages^16^, choosing among visual objects^17^, searching for a target among distractors^18^, navigating complex environments^19^, and even in the selection of possible mates^20^. Performance on visuospatial tasks is enhanced during the inhalation phase of the respiratory cycle^21^. Although the effects of object color on taste^22–25^ and olfaction^25–28^ have been well established, the reverse effects of odorant processing on vision have been less frequently documented. The concurrent processing of the smell of a rose leads to a bias in participants who report the duration of the percept of rivalrous retinal images (i.e., a rose or marker pens) in a continuous flash suppression paradigm^29^, suggesting that the concurrent processing of odorants can influence early visual processing. The odorant congruency effect in binocular rivalry appears to be hemispheric specific for lateralized object presentations during left or right nostril odorant exposure^30^, suggesting that the effect has a spatial specificity. Robinson and colleagues^31^ demonstrated that exposure to a congruent scent enhances the visual saliency of an object presented in rapid serial visual presentation (RSVP) mode as used in an attentional blink paradigm. Congruent odors enhanced detection of the second target (T2) after participants responded to the first target (T1) on trials where T1 was presented 200 ms prior to T2 (i.e., “gap 3” during the attentional blink; see also the work by Colzato and colleagues^32^. However, a recent attempt to replicate this finding has not been successful^33^. Participants inspected images of odorant-congruent object images longer than odorant-incongruent objects^34–36^, suggesting that odorant processing may enhance visual attention to odorant-matching (congruent) objects. It remains to be determined to what extent olfactory processing can influence the early visual processing required for visual search.

In the present research, we explore whether concurrent olfactory processing can affect the performance and response times in a visual search task. To this purpose, we exposed our participants to one of three different odorants while they performed a cued, visual search task. On any given trial, participants unknowingly breathed in either neutral room air or air mildly scented with a strawberry, apple or lemon aroma, and then they were briefly cued to search for a target image of a strawberry, apple or lemon randomly positioned among other fruit distractors. Random couplings between scents and the cued target fruit were presented over trials. Baseline visual search performance was determined by randomly interleaving trials where no odorant was presented to the participants’ nostrils. In odorant trials, the fruit scent could be congruent with the target fruit or incongruent. In the latter case, it was either unrelated to any visual stimulus in the array or matched one of the distractor fruits. Although the odorant was task irrelevant, we could determine an effect of the congruency of odorant-visual object couplings on our participants’ search performance. Our findings point to an odorant-visual object congruency effect that depends on the participants’ baseline visual search performance in the neutral room-air condition: low performers of the visual search task benefit most when the odorant and target are congruent, whereas high performers exhibit the greatest interference when the odorant is incongruent to the target. As such, our results provide support for a role of olfaction in visual search for a food target among other food distractors.

## Results

**Fig. 1:**
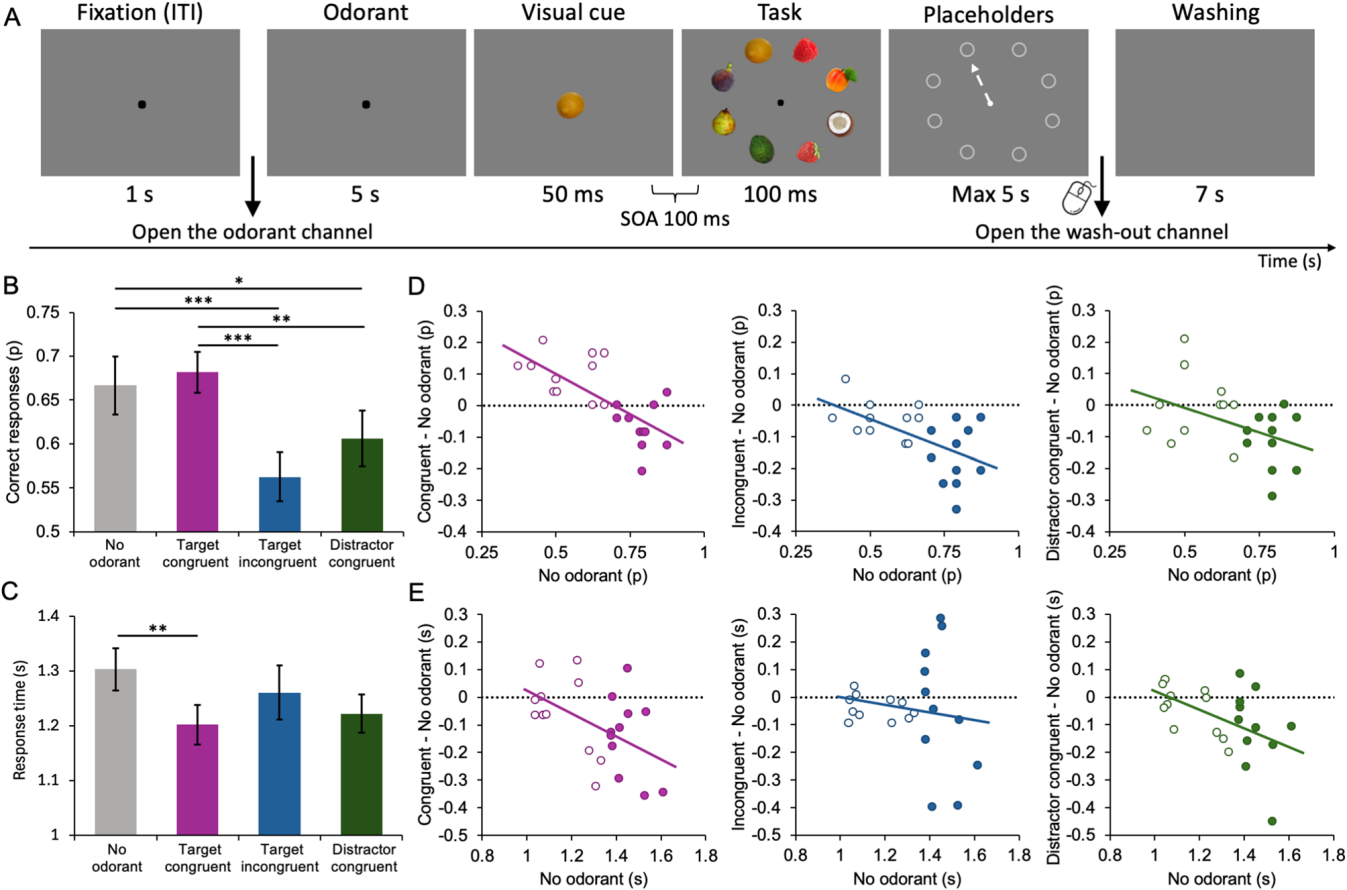
Visual search task procedure and results. (**A**) Experimental design of the visual-search task during concurrent exposure to a congruent, an incongruent or no (neutral room air) odorant. Participants were unaware on a given trial if an odorant was present, and if present whether it would be congruent or incongruent to the cued target and distractors. Fruits, placeholders and the background display are not drawn to scale here (see *Methods*). For illustration purposes, the fruits images are shown with full contrast. The white arrow with dashed shaft signifies the intended cursor movement from the center of the display to the placeholder that contained the target. Following the participant’s response, the display became blank and the olfactometer turned on the wash-out channel containing air flowing through the water bottle with no odorant. The next trial was indicated by the reappearance of the centrally located fixation dot. (**B**) Visual search performance (proportion of correct responses) during concurrent exposure to no odorant or to congruent or incongruent odorants. Post-hoc *t*-tests (Bonferroni correction) show a significant difference between conditions: no-odorant vs. target-incongruent: *t* = 4.5, *p* < 0.001, *d* = 0.7 - *medium* effect size; no-odorant vs. distractor-congruent *t* = 2.96, *p* = 0.043, *d* = 0.5 - *medium* effect size; target-congruent vs. target-incongruent *t* = 5.15, *p* < 0.001, *d* = 0.89 - *large* effect size; target-congruent vs. distractor-congruent *t* = 3.6, *p* = 0.007, *d* = 0.6 - *medium* effect size). (**C**) Response times (in seconds) of correct responses during concurrent exposure to no odorant or to congruent or incongruent odorants. Post-hoc *t*-tests (Bonferroni correction) show a significant difference between no-odorant and target-congruent conditions: *t* = 3.4, *p* = 0.007, *d* = 0.55 - *medium* effect size. (**D**) Individual relative change in visual search performance in the congruent and incongruent odorant conditions is plotted as a function of each participant’s performance in the no-odorant condition. The significant negative correlation indicates that the odorant-visual object congruence effect depends on the participant’s baseline visual search performance level in the no-odorant condition. Left panel: *r* = − 0.7, *p* < 0.001; middle panel: *r* = − 0.56, *p* = 0.007; right panel: *r* = − 0.42, *p* = 0.047. These correlations are not statistically different (Steiger’s Z test: all *p*-values > 0.05, *n.s.*). (**E**) Individual relative change in response times in the congruent and incongruent odorant conditions is plotted as a function of the participants’ response time in the no-odorant condition. Left panel: *r* = − 0.49, *p* = 0.018; middle panel: *r* = − 0.15, *p* = 0.51, *n.s.*; right panel: *r* = − 0.48, *p* = 0.02. The absolute value of the correlation in the incongruent condition (middle panel) is significantly lower than that of the target congruent (Steiger’s Z test: z = − 1,791, *p* = 0.037) and distractor-congruent condition (z = − 1.82, *p* = 0.034). In panels (**D**) and (**E**), open circles represent low performers with baseline scores below the median, whereas filled circles represent high performers with baseline scores above the median in the no-odorant condition. Error bars signify ± 1 standard error of the mean; *p* < 0.05 *; *p* < 0.01 **; *p* < 0.001 ***.

To test the effect of ongoing odorant processing on visual-search performance, we used a four-channel olfactometer. See Supplementary Fig. 1 for an illustration of the apparatus and setup. Participants were exposed to one fruit odorant (lemon, apple, or strawberry) or neutral room air (no odorant) while they searched for an image of a cued-target fruit presented among fruit distractors (Fig. 1A; see *Methods*). The odorant was delivered for 5 s based on prior testing showing that participants could reliably detect the presence of an odorant if presented for at least 3 s (see *Supplementary material* for detection task description - Supplementary Fig. 2). Visual stimuli had a short duration (cue 50 ms, search array 100ms) and desaturated colors to make vision less reliable and allow for a possible effect of olfaction on the visual search task^37^. Nevertheless, such short durations still allow for object recognition^38–40^, also in visual search tasks^7^. For target examples see Supplementary Fig. 3; for distractor examples see Supplementary Fig. 4.

Prior to each trial, participants did not know if an odor would be presented or if so, whether it would be congruent or incongruent to the subsequently presented visual cue. In the baseline no-odorant condition, only neutral room air was delivered to participants during the task. In the *target-congruent* condition, participants breathed in scented air with an aroma congruent to that of the cued target fruit—e.g., when a lemon odorant was presented, and the target fruit was a lemon. In the *target-incongruent* condition, participants breathed in scented air with an aroma that was incongruent with both the cued target fruit and any of the distractor fruits—e.g., a lemon odorant was presented while the target fruit was a strawberry, and no lemon image was present in the search array. Additionally, we included a target-incongruent, *distractor-congruent* condition, in which the scented air contained an aroma incongruent with that of the target but congruent with one of the fruit distractors—e.g., a lemon odorant was presented while the target fruit was a strawberry, but a lemon image was present among the distractors. Despite the fact that the odorant was task irrelevant, we expected to find a search facilitation due to visuo-olfactory congruency and a potential disruption effect due to incongruency, which should be even more pronounced when the task-irrelevant odorant matches one of the visual distractors.

We first explored the effect of odorant-visual object congruency of visual search performance. Figure 1B depicts the proportion of correct responses for the four possible odorant-visual object couplings. The results indicate that search performance is significantly modulated by the congruency of the odorant-visual target coupling. An ANOVA analysis indeed indicated a highly significant main effect of congruency on the search performance (F_3,63_ = 11.88, *p* < 0.001; *η*^2^ = 0.36 - *large* effect size). There is a highly significant difference between the conditions where the odorant is congruent or incongruent to the searched-for fruit target (significant post-hoc pairwise comparisons are reported in the caption of Fig. 1B).

An analysis of the errors made during the visual search task indicates that the participants most likely (approx. 47% of all errors) selected a placeholder that had contained a distractor (e.g., raspberry) with a color similar to that of the visual target (e.g., strawberry), with the remaining errors evenly distributed over the other alternatives that mismatched the target on color and odorant (χ^2^(12) = 48.07, *p* < 0.001). The odorant appeared to play a lesser role in determining the selection of a placeholder location which the participants erroneously thought to have contained the target. The distribution of errors did not depend on the odorant presented (χ^2^(6) = 7.8, *p* = 0.25, *n.s.*), but it did depend on the condition of odorant-visual object congruency (i.e., target-congruent, target-incongruent, distractor-congruent; chi^2^-test = 22.27, df = 6, *p* < 0.001).

Our participants were instructed to move the cursor to the placeholder containing the target, using the computer mouse. Figure 1C depicts the response times of the participants when they performed the visual search task correctly. Here, there is a significant main effect of target visual-olfactory congruency (F_3,63_= 4.49, *p* = 0.006; *η*^2^ = 0.18 - *large* effect size) on response times: when the aroma of the scented air was congruent to that of the fruit target, participants required less time to guide the cursor to the target placeholder compared to response times in the no-odorant condition (significant post-hoc pairwise comparisons are reported in the caption of Fig. 1C). The incongruent conditions did not significantly differ from the no-odorant condition.

Importantly, additional analyses showed that visual-olfactory congruency effects on search performance and response times do not differ across different visual targets and fruit scented-odorants (strawberry, apple, lemon; see Supplementary Fig. 5).

Because visual search efficiency can vary as a function of visual field position^41^, we examined potential location effects by testing whether the quadrant in which the target appeared (upper-left, upper-right, lower-left, lower-right; see *Methods*) influenced participants’ performance or response times in the baseline condition. The results indicate that the probability of a correct response was independent of target position (χ^2^(3) = 5.16, *p* = 0.16, *n.s.*). Likewise, a one-way ANOVA on response times revealed no significant effect of quadrant (F(3, 522) = 2.28, *p* = 0.078, *n.s.*). Overall, these results indicate that search efficiency in our task was largely unaffected by target spatial position and did not reflect asymmetries between upper and lower or left and right visual fields.

In a post-experimental recognition test, participants were required to name the odorants they believed they had smelled (see *Methods* for details), to assess whether differences in congruency effects could be related to the explicit recognition of fruit-scented odorants. Response scoring revealed that 14 out of 22 participants correctly named all three odorants, six recognized only two out of three, and two identified only one. Importantly, no significant correlations emerged between odorant recognition scores and visual search performance in the different conditions (Spearman’s rank correlation coefficient; all *p*-values > 0.05, *n.s.*). These results suggest that the olfactory–visual congruency effect is likely implicit and does not depend on conscious perception of the odorants.

Our paradigm allowed for a post-hoc analysis of the correlation between search performance in the neutral (no odorant) trials to those performance levels exhibited in the trials with olfactory stimulation. Figure 1D presents a scatterplot of the individual participant’s performance enhancement (positive values) or impairment (negative values) as a function of his or her respective performance in the baseline condition where neutral room air was delivered by the olfactometer during visual search. There is a highly significant negative correlation between the differential performance of the participants and their baseline (no odorant) performance (median of 0.69). For the target-congruent condition, these results indicate that low visual-search performers (baseline values below the median) benefited from the congruency of the delivered odorant, whereas high performers (baseline values above the median) showed no improvement or even lower performance (Fig. 1D—left panel). In contrast, with incongruent odorant–visual pairings, high performers exhibited a stronger impairment relative to trials without odorants, compared to low performers (Fig. 1D—middle and right panels).

In a similar fashion, we could explore post-hoc correlations between response times in the different olfactory conditions compared to the neutral (no odorant) condition (median of 1.33 s). Figure 1E presents a scatterplot of the individual participant’s response time reduction (negative values) or elevation (positive values) as a function of his or her respective response time in the baseline condition. The negative correlation in the congruent condition indicates that the reductions in response times for congruent cross-modal odorant cueing were more pronounced in slow responders (high baseline, longer response times), while fast responders (low baseline) did not show a marked facilitation (Fig. 1E—left panel), again linking the cross-modal congruency effect with the individual participants’ search efficiency in the baseline (no odorant) condition. Note, however, that in conditions of incongruent couplings slow responders also exhibited a more pronounced reduction in response times compared to faster searchers (Fig. 1E—middle and right panels).

**Fig. 2:**
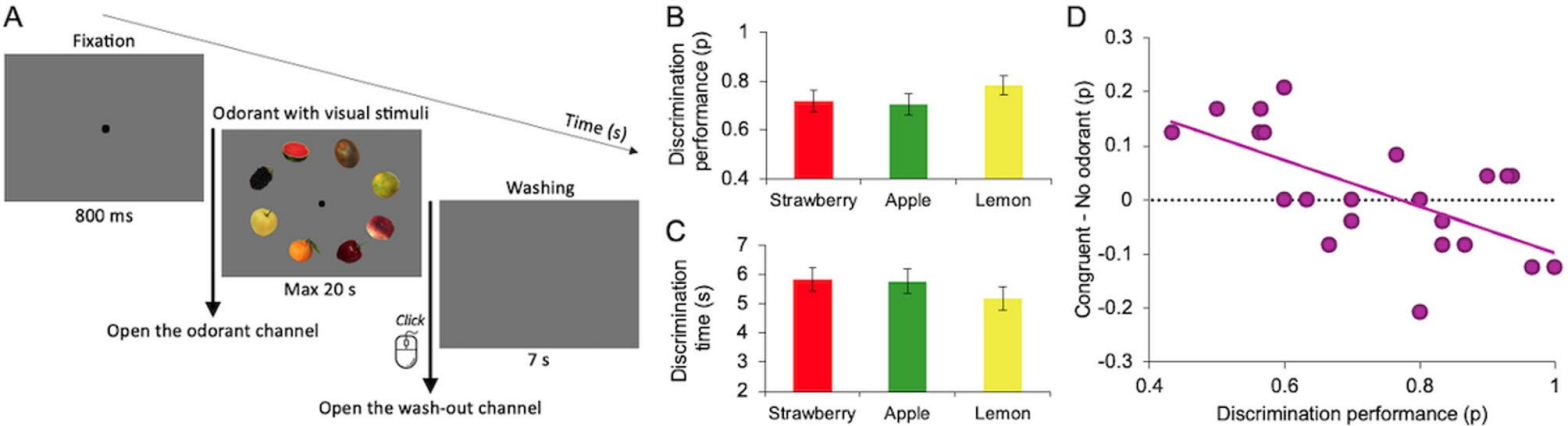
Discrimination task procedure and results. (**A**) Experimental control task to determine participants’ ability to correctly discriminate the aroma of the target fruit among fruit distractors. Fruit objects and the background display are not drawn to scale here (see Methods). For illustration purposes, the fruit images are shown with full contrast. (**B**) Discrimination performance, presented as a proportion of correct responses, for the three odorants used in the main experiments. (**C**) Discrimination time of correct responses for the three odorants used in the main experiments. Error bars signify ± 1 standard error of the mean of the results of *n* = 22 participants. No significant differences were evident between the three fruit scents used in the experiments. (**D**) Correlation between the relative change in visual search performance in the congruent odorant versus the no odorant condition as a function of each participant’s performance in the discrimination control task (*r* = − 0.64, *p* = 0.001). Points represent data from individual participants.

Figure 2A depicts the procedure of the discrimination control task we used to determine the extent to which our participants could correctly discriminate among the different fruit aromas (see Methods). In the discrimination task, one odorant channel opens (scent of lemon, strawberry, or apple), and participants had to select the fruit matching the perceived aroma amongst eight visual stimuli. This task was conducted after the main visual search task to avoid influencing the participant’s behavior in the main experiment. Figure 2B shows discrimination performance (proportion of correct responses) for the three fruit-scented odorants, while Fig. 2C presents the average response times (correct responses only). On average, participants correctly discriminate the fruit scent in 73% of trials in 5.6 s. ANOVA analyses show that there were not any significant differences between the three fruit scents with respect to the participants’ odorant discrimination performance (F_2,42_= 2.4, *p* = 0.102, *n.s.*) and response times (F_2,42_= 2.1, *p* = 0.12, *n.s.*).

Figure 2D shows the relative effect of the congruent odorant during visual search plotted as a function of the discrimination performance in the control discrimination task. The negative correlation is highly significant (*r* = − 0.64, *p* = 0.001). Two aspects of the results need to be noted. First, the participants varied considerably in their ability to discriminate between the fruit aromas presented randomly to them via the olfactometer. While some participants performed nearly perfectly, others indicated discrimination performance levels that were above guessing levels but still low. Second, the participants who had more difficulty discriminating between the different fruit odors (value on x-axis) benefited most when these odorants were congruent to the searched-for target fruit (value on y-axis). Interestingly, participants who easily discriminated between the different fruit odorants showed little or no benefit of the congruent odor-visual couplings in the search task. Some even demonstrated lower performance when the odor they were exposed to was congruent to the cued target fruit. Note that the relative changes in visual search performance for the target incongruent and distractor congruent conditions do not correlate significantly with the odorant discrimination performance (Pearson correlation coefficients; all *p*-values > 0.05, *n.s.*; not shown in Fig. 2).

## Discussion

Our findings point to a task-irrelevant, multisensory congruency effect during speeded visual search tasks. Exposure to an odorant together with a cued visual search target (i.e., a piece of fruit) presented among similarly shaped objects (exemplars of other types of fruit) led to significant differences in search performance (Fig. 1B) depending on the implicit odorant-visual target congruency of the scented air breathed in orthonasally by the participants. Furthermore, correlational analyses revealed that the effect of exposure to the fruit-scented air during visual search depended on the performance levels of the participants in the baseline (no odorant) condition. Poor performance on the visual search task (i.e., values below the median no-odorant baseline level) was associated with a larger benefit from congruent aroma-visual couplings (Fig. 1D—left panel). This may reflect the fact that participants who struggle under unimodal (no-odorant baseline) conditions benefit more from implicit multisensory cues, possibly because a congruent olfactory stimulus provides an external guiding signal that reduces search demands and helps focus attention on the relevant object. By contrast, individuals with high visual search efficiency may not benefit as much from congruent odor cues, as their visual search performance is already close to the ceiling level, leaving little room for any further improvement; in such cases, the implicit olfactory signal may even be redundant. On the other hand, the impairment in search performance observed with incongruent aroma-visual couplings was more pronounced in these high-performing participants (Fig. 1D, middle and right panels). High performers may have been more strongly focused on the visual task, making them more susceptible to distraction from conflicting multisensory input, whereas low performers—being less efficient in the visual search—may have been less affected by any crossmodal interference. Such individual differences in search efficiency have been well documented^42^.

Congruent cross-modal cueing speeds up visual search, with participants finding the target faster in the presence of a congruent odorant (Fig. 1C). This reduction in response times was stronger in individuals who were slower in the baseline (no-odorant) condition (Fig. 1E, left panel), suggesting that congruent odor cues mainly benefit participants with low visual search efficiency, while offering little or no advantage to fast performers. Surprisingly, slower participants also showed reduced response times in the incongruent conditions (Fig. 1E, middle and right panels). This counterintuitive finding may indicate that, for these participants, the presence of any additional sensory cue—even when incongruent—acts as a general alerting signal, increasing arousal and thereby facilitating faster responses^43^.

The congruency effect on performance also depended on the ability of participants to discriminate between the fruit scents presented (Fig. 2). Participants with lower odor discrimination ability appeared to benefit from the presence of a congruent olfactory cue, whereas those with higher discrimination ability showed no performance facilitation, sometimes even a disruption. Such individual differences might reflect differences in the processing of multisensory task-irrelevant cues. Individuals with good odor discrimination may analyze the olfactory signal more deeply, treating it as an additional source of information that sometimes interferes with visual processing. In contrast, poor discriminators may not fully process the odor and therefore rely more heavily on any external aid that implicitly facilitates visual search, even if it is not fully decoded^44^. These differences might also depend on the different brain regions engaged in the task. Gottfried and Dolan^45^ found an association between behavioral olfactory-visual congruency effects and enhanced fMRI activations in anterior hippocampus and rostral medial fronto-orbital cortex. Further fMRI evidence for a graded effect of visual categories (oranges vs. peanuts) and a continuous spectrum of odorants (stepwise dilution mixtures of the respective odorants) points to a mediating role of the superior frontal gyrus in task difficulty associated with olfactory (piriform cortex) - visual (fusiform cortex) interactions^46^. Olfactory-visual congruency effects on functional brain connectivity have also been reported for semantic categories (healthy vs. unhealthy foods^47^. Evidence for individual differences in cross-modal cueing in an audiovisual matching task has been reported by Plank and colleagues^14^, who found with fMRI a significant correlation across participants between the activation in inferior frontal gyrus and performance in a spatial auditory location task^14^. Such brain areas may support the neural processing of cross-modal stimuli during demanding search tasks and the extent of this engagement of different brain regions may vary across participants.

Importantly, our post-experiment recognition test provided an indirect measure of the conscious perception of the odorants. Although participants differed in the number of odorants they were able to correctly identify, these differences were not related to visual search efficiency in the different congruency conditions. This finding suggests that the observed olfactory–visual congruency effect is not driven by conscious perception or explicit knowledge of the odorants, but it rather reflects an implicit crossmodal influence.

Our present findings support the role of olfactory processing in the visual search of characteristic food items. Individuals searching for a lemon among apples, oranges and mangos may benefit from concurrent exposure to air carrying the scent of lemons. As such, our findings provide support for the role of olfaction in human spatial cognition^19^. The dependency we found between the amplitude and sign of the congruency effect on the individual participant’s search performance level suggests that near-threshold stimulation might be best suited to reveal the effects of congruent multisensory olfactory-visual integration^48^. The benefits of congruent cross-modal stimulation therefore appear to depend on each individual’s ability to perform a speeded visual search for a target among similarly shaped distractors.

Attention can be directed to a visual target by cross-modal cues. The central presentation of the target in a visual search task can be compared to endogenous cueing as used in similar speeded visual tasks^49^. In our paradigm, the processing of a brief (50 ms) centrally presented target to be searched for among similarly sized and shaped distractors may be influenced by the prior exposure to a congruent or incongruent cross-modal cue, such as the aroma of a fruit. Such cross-modal cueing is by definition endogenous, since it is task-irrelevant and spatially non-informative. Exposure to pleasant odorants can heighten arousal and direct attention to exemplars of the cued category (e.g., exposure to the scent of lemon primes the higher-order processing of that specific type of food category). Pleasant odorants appear to attract visuo-spatial objects, whereas unpleasant odorants have a repulsive effect^50^. Our present findings show that participants with long baseline response times showed reductions of 25% or more when exposed to congruent odorants. Such effects point to the “benefits” of valid cues^51^ that direct attention to a semantically matching target object presented among distractors, thereby enhancing its saliency^13^. By contrast, incongruent odor–visual pairings acted as invalid cues in our search paradigm and they impaired performance, particularly in participants who were otherwise efficient in visual search. The association between the magnitude and sign of the odor-visual object congruency effect and individual performance levels suggests that cross-modal cueing serves to increase signal-to-noise ratios in the sensory processing of stimuli in speeded tasks that demand quick decisions.

## Limitations of the present study and outlook

As with every study, there are clear limitations with respect to our ability to generalize our results to other visual functions. A speeded visual search is a demanding task, which forces the participant to choose quickly one among many alternatives. On trials where they are uncertain, we encouraged our participants to guess the most likely alternative. There is also the possibility that such guesses will be biased by response tendencies. Future research should explore in more detail the effects of cross-modal cueing on response bias^52^ in tasks with high levels of stimulus uncertainty^53^.

A further consideration relates to our choice of mouse movements as a behavioral measure. We adopted mouse-based response because the task involved eight possible response options, rendering key-press responses less suitable. However, we acknowledge that this approach may introduce between-subject variability related to motor control or movement fluency. In this regard, it is important to note that our measure reflects *response time* rather than *reaction time*, as it includes both decision and motor components. A valuable option for future research would be to use eye-tracking recordings to obtain more direct and fine-grained measures of visual search dynamics.

Another limitation concerns the sample size, as our study included a moderate number of participants, which may have reduced overall sensitivity to detect further differences across conditions. For example, the distractor-congruent condition did not lead to more frequent erroneous selection of the distractor that matched the scent presented during the trial or longer response times compared to the target-incongruent condition. While some studies tested larger samples^33,34^, our sample size is in line with much of the published literature on olfactory–visual interactions^29–32,35,36^. In addition, the predominance of female participants represents a potential source of bias, although the literature on gender differences in olfactory perception is not entirely consistent. Indeed, some large-sample studies report no gender differences in smell detection ability^54^ or olfactory identification^55^, while others find enhanced performance in women^56^. Nevertheless, we acknowledge that this gender imbalance may limit the generalizability of our findings.

Future research should aim to replicate and extend our findings with larger, more gender-balanced samples, and using tasks designed to minimize the influence of motor-related variability and response biases under uncertainty, thereby allowing for a more precise characterization of cross-modal olfactory–visual interactions.

## Conclusions

Our findings indicate that the concurrent processing of an odorant can influence a person’s performance in a speeded visual search paradigm. The effect of concurrent odorant processing, although task-irrelevant for visual search, appears to depend on the ability of our participants to perform the speeded search task: poor search performers benefit most from the presence of congruent odors, whereas good search performers are impaired most by exposure to incongruent odors. Such a dependence on search performance suggests a possible role of a task irrelevant cross-modal cue (e.g., the exposure to the scent of lemon) to direct the participant’s attention to the subsequently cued visual target (e.g., an image of a lemon). Processes related to visual attention may benefit from cross-modal cues related to the semantic categories of potential targets. Search for a lemon, for example, among other fruits appears to benefit from prior congruent olfactory information. Such multimodal integration of olfactory with visual cues might play a role in our food choices in everyday situations. Further research would need to better define the nature of such higher-order cognitive processes in real-world scenarios.

## Methods

### Participants

A total of 22 healthy adult volunteers (20 females, 2 males; mean age = 28.7 years, SD = 12 years) participated in the study. All participants were pre-screened for psychiatric and neurological disorders and were not taking psychoactive medication at the time of the study. All selected participants had normal or corrected-to-normal vision. No participant reported themselves as having a history of anosmia. All but three were right-handed. Participants were unaware of the aims of the experiment, and they gave written informed consent before participating in the study. The study has been approved by the ethics committee at the University of Regensburg (Proposal number: 25-4084-101) in accordance with the Declaration of Helsinki.

Recruitment was limited by several practical constraints. Eligible participants had to be non-smokers, report no recent colds, and have not contracted COVID in the past year. Additionally, some individuals who were initially recruited withdrew before data collection, after the practice trials, because they felt uncomfortable with the nasal cannula, were irritated by the airflow in their nostrils, or reported side effects such as transient headaches or mild stomach discomfort caused by the odorant delivery and/or prolonged use of the chinrest.

### Apparatus

All stimuli were programmed on a Dell OptiPlex 5090 computer running Windows 10 with Matlab 2015, using Psychophysics Toolbox. The experiment was displayed on a gamma-corrected Philips monitor (1920 × 1080 pixels resolution, 49.5 × 29.5 cm, 60 Hz refresh rate), subtending 49.5 × 29.5° degrees of visual angle at a 57 cm viewing distance. All experiments were carried out in a completely darkened room and observers’ heads were supported by a chin- and forehead-rest. Participants’ manual responses were provided on a standard Dell keyboard. JASP software (JASP team, Version 0.19.3, Release 2025, https://jasp-stats.org/) was used for statistical analyses.

A Canon EOS M50 camera with an EF-S 55–250 mm f/4.5–5.6 lens was used to photograph fruits. Adobe Photoshop 2020 and Photoscape X software were utilized to modify images to match them in size and contrast.

The computer-controlled, air-dilution olfactometer was a custom-made device constructed in the workshop of the University of Regensburg, with a design inspired by Johnson and Sobel^60^. It consisted of four bottles; three glass Woulfe’s bottles were filled with 500 ml water and 1.2 ml water-based odorant (see below). The fourth bottle contained only water. The water used was low-residue mineral water (“Schloss Quelle Naturelle” mineral water). The air stream with positive pressure was delivered to the participants via a nasal cannula and a pair of prongs (Tiga-Med. GmbH, Greiz, Germany) exposing both nostrils to one of three different odorants or neutral air depending on which bottle was open. The air flow was approximately 8 L/min (corresponding to that used in the study of Han and colleagues^61^, which was adjusted in pilot work to provide sufficient orthonasal odorant exposure without the participants actively sniffing. See Supplementary Fig. 1 for an illustration of the apparatus and setup.

### Stimuli

Visual stimuli consisted of photographs of fruits. The fruits were photographed under natural lighting conditions at midday, ensuring consistent illumination across all items. Images of fruits with defects or those with particularly recognizable colors and/or shapes (e.g., bananas, which would differ too much from the round shape of other fruits) were discarded. All selected photographs were modified to remove the background, crop the fruit silhouettes, and standardize the color and lighting. Each fruit was then placed at the center of a gray square (greyscale value = 127) with dimensions of 320 × 320 pixels. Final images were standardized to a resolution of 72 pixels per inch. Twenty-eight different fruits were selected. For each fruit, multiple specimens were photographed from different angles, both whole and cut (peeled or halved). From these, at least 10 different photos were chosen, including images of different specimens of the same fruit, different angles of the same specimen, and variations in its state (whole, peeled, or cut in half). See Supplementary Fig. 3 for examples of our three target fruits—strawberry, apple, and lemon—which were used as “cues” in the visual search task. All other fruits were used only as distractors; see Supplementary Fig. 4 for one example of each.

Olfactory stimuli consisted of three odorants: strawberry, apple, and lemon (ExtraChem, GmbH, Bielefeld, Germany). The ingredients included triacetin, citral (lemon), propylene glycol, benzyl acetate (strawberry), ethoxylated castor oil, fructone (green apple), respectively for each odorant, and benzyl alcohol^62^ (personal communication from ExtraChem, GmbH). These substances are all approved for use in the food and cosmetic industries. They contain no harmful ingredients. The concentration of odorants was determined in pilot studies. To achieve odorant detection within 5 s and to achieve a discrimination threshold around 70% correct, we added 1.2 ml of odorant liquid to 0.5 L of bottled water, yielding a concentration of 0.0024 for all three odorants used in the experiments described in this study.

### Procedure

The experimenter prepared the olfactometer and covered the bottles to ensure that the participant remained blinded to the type and number of odorants used. They then put on the nasal prongs and sat comfortably in front of a screen, resting their head on a chin rest. We instructed all participants to keep their mouths closed and breathe through their noses. The entire experimental session consisted of three different, sequential tasks: a detection task (preliminary task), a visual search task (experimental task), and a discrimination task (control task), followed by a final recognition test.

Before the main experiment, preliminary testing on an independent sample was conducted to select the most suitable odorants and determine their optimal concentrations. These precautions ensured that each odorant could be detected within a maximum of five seconds after the respective channel was opened by the olfactometer. Additionally, a seven-second wash-out period was determined to be sufficient for the neutral airflow to fully eliminate the previous odor.

#### Detection task

Before performing the main experiment, participants completed an odor detection task designed to assess their ability to perceive the odorants within the expected timeframe (see Supplementary Fig. 2). Each trial began with the presentation of a blank screen for 500 ms, with the background consistently set to a medium gray (RGB value = 127). Immediately afterwards, the odorant channel was opened, allowing the scented air to flow into the participant’s nostrils. Participants were instructed to press the right arrow key as soon as they perceived an odor, with a maximum response time of 15 s. Once the detection phase ended, the wash-out phase began, during which the wash-out channel was opened for a maximum of 15 s, allowing the odor to dissipate. Participants were instructed to press the spacebar as soon as they could no longer perceive the odor.

This task allowed for the measurement of both detection time, which corresponded to the time required to perceive the odor after the channel was opened, and washing-out time, which was defined as the time needed for the odor perception to disappear following the activation of the wash-out channel. The goal of this task was to confirm that the olfactory presentation times selected for the main experiment, consisting of five seconds of odorant presentation and seven seconds of wash-out, were appropriate for our participants. The detection task included six initial test trials, which allowed participants to familiarize themselves with the inhalation of the odorants, followed by 30 experimental trials. Each odorant was presented on 10 trials each, with the order randomized across trials. This task lasted approximately 7 min, after which participants took a five-minute break before proceeding to the main experiment.

#### Visual search task

The main experimental task consisted of a visual search paradigm with concurrent odorant presentation (see Fig. 1A). Each trial began with the presentation of a small black fixation point (10 pixels), displayed at the center of the screen for one second. This was followed by the odorant presentation. Participants were unaware of whether an odorant was present on a given trial, and if present, they did not know what type of odor it was nor whether it would be congruent or incongruent with the visual target. The fixation point remained visible, and participants were instructed to maintain fixation at all times. After 5 s of odorant delivery, a target fruit image appeared at the center of the screen for 50 ms (3 frames). The specific target exemplar was randomly selected at each trial. Following a stimulus onset asynchrony of 100 ms, a visual array consisting of eight fruit images was displayed. The stimuli were arranged in a circular layout, with all images equidistant from the center at an eccentricity of 7 degrees. The positions of the stimuli were defined by eight polar angles (30°, 60°, 120°, 150°, 210°, 240°, 300°, and 330°), avoiding cardinal axes to reduce positional biases. Each stimulus measured 64 pixels in size, corresponding to a visual angle of 2 degrees. The target fruit was always present in the array, accompanied by seven distractors. The distractor stimuli were randomly selected at each trial, ensuring that the same fruit did not appear more than once within the same array.

Preliminary testing had indicated that under conditions of full color saturation and longer stimulus durations (150 ms cued target, 200 ms search array), participants achieved an accuracy level of approximately 90% in the visual search task. To increase task difficulty and better observe potential olfactory facilitation or inhibition effects on the visual search^37^, the stimuli were presented at 50% color saturation and displayed for shorter periods (50 ms cued target, 100 ms search array). Such short durations still allow object recognition^38–40^, and are also used in visual search tasks^7^.

At the end of the stimuli presentation, the fruit images disappeared, leaving only placeholders on the screen while the participant’s response was awaited. Participants were given a maximum of five seconds to respond. The mouse cursor appeared at the start of this response phase, and participants were instructed to search for the target fruit among the distractors and move the cursor to its corresponding location as quickly as possible. Once the participant clicked on a location or after the maximum response time elapsed, the wash-out channel was opened, allowing the odor to dissipate before the next trial began. The onset of the following trial was indicated by the reappearance of the central fixation dot.

The experiment consisted of a total of 96 trials, divided into two runs with 48 trials per block. Each odorant (strawberry, apple, and lemon) was presented in 24 trials, while 24 additional trials were conducted in a baseline condition with no odorant, in which only neutral room air was delivered. Odorants and the no-odorant condition were presented in random order across trials. In the odorant-present trials, three different conditions were included. In the target-congruent condition, the olfactory stimulus corresponded to the visual target, such as when a lemon odorant was presented and the target fruit was a lemon. In the target-incongruent condition, the olfactory stimulus did not match the visual target, and the corresponding fruit was absent from the distractor set, as in the case where a lemon odorant was presented, but the target fruit was a strawberry and no lemon image was present in the search array. In the distractor-congruent condition, the olfactory stimulus did not correspond to the visual target, but the corresponding fruit was included among the distractors, such as when a lemon odorant was presented, the target fruit was a strawberry, and a lemon was present among the distractor images.

Each experimental run lasted approximately 12 min, with a five-minute break between the two blocks of trials.

#### Discrimination task

After a 5-minute break, participants performed a discrimination task, which served as an experimental control task to assess their ability to correctly identify the aroma of the target fruit among fruit distractors (see Fig. 2A).

Each trial began with the presentation of a central fixation point (10 pixels) for 800 ms, followed by the release of an odorant through the olfactometer. The odorant remained present for a maximum duration of 20 s, during which participants had to identify the corresponding fruit by clicking on one of the fruit images displayed on the screen. As in the experimental task, the stimulus array consisted of multiple fruit images, including the target fruit and various distractors (same size and eccentricity as described above). Participants were instructed to make their selection as quickly and accurately as possible. Once the response was registered or the maximum time elapsed, the wash-out phase commenced, during which the wash-out channel was opened for seven seconds to ensure the complete dissipation of the odor before the next trial.

The discrimination task included a total of 30 trials, with each odorant (strawberry, apple, and lemon) presented in 10 trials, randomized across the experiment. The total duration of the discrimination task was approximately 7 min.

#### Recognition test

Finally, participants completed a post-experimental recognition test, in which they were asked to name the fragrances they believed they had smelled. This test was included to evaluate participants’ explicit awareness of the different fruit-scented odorants and could then inform whether individual differences in visual search performance is related to the explicit recognition (i.e., conscious perception) of the olfactory stimuli.

The whole experimental session lasted about 1 h. The number of trials within each task was intentionally kept low to avoid fatigue and any overstimulation of the olfactory receptors, which could cause discomfort to the participants.

### Data processing and statistical analyses

#### Detection task

On each trial, the time elapsed from the opening of the odorant channel to the participant’s response was recorded as the *detection time*. The time elapsed from the opening of the wash-out channel to the participant’s response was recorded as the *washing-out time*. For each participant, the average detection and washing-out time for each odorant was calculated, excluding trials where no response was given (i.e., when the maximum time was reached without a key having been pressed — only 5 trials out of 660 total, < 1%, were excluded). Then, the data were averaged across participants, obtaining mean detection and washing-out times for each odorant (see Supplementary Fig. 2B and 2 C). Data were normally distributed (all Shapiro-Wilk tests yielded no significant *p*-values), so they could be compared using one-way repeated measures ANOVAs (factor: *fruit scent*, with three levels: strawberry, apple, lemon).

#### Visual search task

For each trial, we recorded the *response time*, defined as the time elapsed from the search array presentation and the moment the participant clicked the left mouse button on one of the eight placeholders. To identify the movement onset and prevent recording minor hand tremors on the mouse, we set a threshold of at least 15 pixels of displacement. We then recorded the X and Y mouse coordinates to determine the final position of the mouse click. In additional analyses, we examined movement onset times separately and found a constant delay of approximately 70 ms relative to the final click. Because participants could start moving the mouse before making a decision and sometimes “wander” before clicking, we considered the exact time of the mouse click to provide a more reliable measure of search performance.

To determine the participant’s choice, we applied a 1-degree tolerance around each placeholder (32 pixels) and ensured that there were no ambiguous intermediate positions between stimuli. Thus, we could calculate the number of correct responses and obtain the individual search *performance.*

Data from the two runs was pooled together for the analyses (after checking that there were no significant differences in search performance across the two blocks). Only 0.2% of trials (5 out of 2112 total) were not included as no response was given (i.e., the maximum time allowed was reached without any mouse click). Data followed a normal distribution, as confirmed by Shapiro-Wilk tests (all *p*-values > 0.05, not significant for all conditions). For each participant, we compared performance and average response time (correct responses only) across the different odorant conditions using a one-way repeated measures ANOVA (factor: *congruency* = no-odorant, target-congruent odorant, incongruent odorant, distractor-congruent odorant). ANOVA effect size was estimated using eta-squared statistics (*η*^2^). Pairwise comparisons were done with post-hoc t-tests (Bonferroni corrected), and the size effect of differences between conditions was evaluated by Cohen’s *d* statistics.

To assess potential effects of target position, we grouped the eight possible target locations in the search array into four quadrants (upper-left, upper-right, lower-left, lower-right; two stimuli per quadrant) and analyzed performance and response times in the baseline condition. We then compared the proportion of correct responses across quadrants using a chi-square test of independence. Differences in response times were evaluated with a one-way ANOVA (factor: *quadrant*, four levels).

To test whether different fruit scents have an influence on the odorant-congruency effect we also analyzed performances and response times with a three-way repeated measures ANOVA (factor: *congruency*, with three levels: target-congruent, target-incongruent, distractor-congruent; factor: *visual target*, with three levels: strawberry, apple, lemon; factor: *fruit scent*, with three levels: strawberry, apple, lemon). Results are reported in the Supplementary Fig. 5.

Pearson correlations were computed between the proportion of correct responses and response times (correct trials only) for the conditions with target-congruent, target-incongruent and distractor-congruent odorant-visual object couplings with the respective values for the no-odorant baseline condition (Fig. 1D,E). The differences in correlation strength between conditions were assessed using Steiger’s Z test^63^.

As our visual search task was challenging and led to many participant errors, we performed an error analysis. We used contingency tables and chi-square statistics to determine if the distribution of these errors were evenly distributed over the odorant-visual object congruency, the odorant presented on the trial and the error type (i.e., distractor matched target in color or odor but mismatched on other stimulus dimensions). This analysis could provide us information about which stimulus features led to these errors.

#### Discrimination task

For each participant, the probability of correctly identifying the odorant was measured, i.e., the *discrimination performance* for each odorant. Also, *discrimination times* were measured as the interval between odor onset and participant selection. Movement thresholds (in pixels) for response time and position were the same as in the experimental task. Only 0.2% of trials (2 out of 660 total) were not included as no response was given (i.e., the maximum time allowed was reached without any selection). Data followed a normal distribution, as confirmed by Shapiro-Wilk tests (all *p*-values > 0.05, not significant for all conditions).

Discrimination performance and response time (correct responses only) across odorants was compared using a one-way repeated measures ANOVA (factor: *fruit scent*, with three levels: strawberry, apple, lemon). Individual discrimination performance was then averaged across odorants to obtain a single discrimination ability score, which was subsequently correlated with the congruency and incongruency effects measured in the main task (Pearson correlation).

#### Recognition test

Participants’ responses in the post-experiment test were evaluated by scoring how many of the named fragrances were correctly identified (ranging from 0/3 to 3/3). Individual recognition scores were correlated with the participants’ performance on the main visual search (non-parametric Spearman’s rank correlation coefficient), to assess whether conscious perception of the odorants was related to congruency and incongruency effects.

## Supplementary Information

Below is the link to the electronic supplementary material.

Supplementary Material 1

## Data Availability

The datasets analyzed during the current study are available in the Zenodo repository: 10.5281/zenodo.15077547.
