# Supplementary material for: Visual search performance depends on the congruency of olfactory sensations
Source: Sci Rep. 2025 Oct 31;15:38116. doi: 10.1038/s41598-025-25995-1 (PMC12578794; doi:10.1038/s41598-025-25995-1)
Supplement: Supplementary file 1 — Supplementary Material 1 [file 41598_2025_25995_MOESM1_ESM.pdf]

Visual search performance depends on the congruency of olfactory sensations

Serena Castellotti, Marija Soldo, Tina Plank, Maria Michela Del Viva, and Mark W. Greenlee

SUPPLEMENTARY MATERIAL

Apparatus and set-up

Our olfactometer was inspired by the design published by Johnson and Sobel[60]. **Supplementary Figure 1** shows the apparatus together with the arrangement of the participant's station.

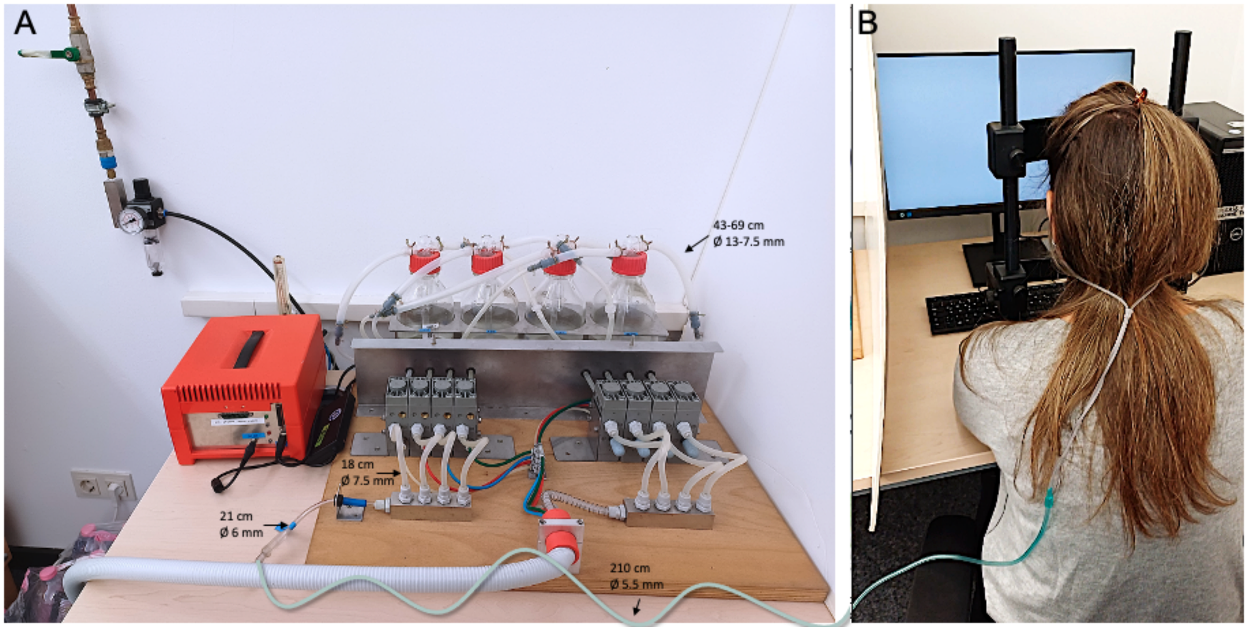

Supplementary Figure 1. Apparatus and set-up. (A) The photo shows the olfactometer with its four bottles (containing water and/or water-based odorant), the tubing system that directs the air stream under positive pressure, and the valve device that opens and closes the channels under software control. The length and diameter of tubes are reported in the picture. **(B)** The photo shows a participant positioned on the chin rest, while wearing the nasal cannula and looking at the screen. For illustrative purposes, the tube connecting the two panels has been manually added to the figure.

Detection task

Supplementary Figure 2A shows the detection task procedure, performed before the visual search experiment. **Supplementary Figure 2B** presents the average detection time, that is the time required after the odorant channel was opened and when the participant pressed a key to signify the presence of a fruit scent. Participants required, on average, 2.9 seconds (± 0.2 SE across odorants) to detect the presence of a fruit scent (strawberry = 2.92 s ± 0.24; apple = 2.66 s ± 0.19; lemon = 3.06 s ± 0.25 SE across participants). ANOVA shows that there were not any significant differences between the three fruit scents with respect to the participants' detection response times ($F_{2,42}$= 2.6, *n.s.*). **Supplementary Figure 2C** presents the average washing-out time, that is the time required after the odorant channel was closed and when the participant pressed a key to signify the absence of a fruit scent. ANOVAs show that there were not any significant differences between the three fruit scents with respect to the participants' washing-out times ($F_{2,42}$= 2.02, *n.s.*). These results allowed us to confirm with our participants that the timing used in the main experiment (i.e., 5 seconds of odorant exposure and 7 seconds of washout) is appropriate to ensure that the odorant is perceived and that there is sufficient time between odorants to clear the nostrils adequately of any residual odorant.

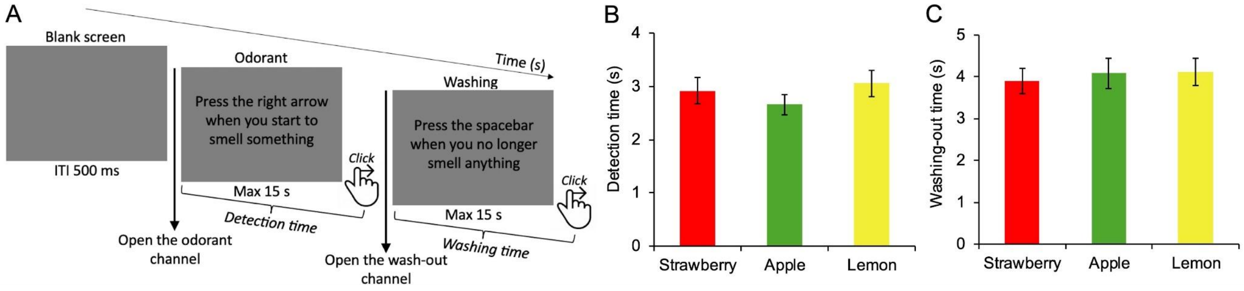

Supplementary Figure 2. Detection task procedure and results. (A) Experimental design of the detection task employed to assess participants' ability to perceive the odorants within the expected timeframe. **(B)** Detection time: stimulation times (in seconds) required for the participants to detect the presence of an odorant are shown, i.e., the single choice (odorant present) response delay between the opening of the odorant channel and the key press given by the participant. **(C)** Washing-out time: stimulation times (in seconds) required for the participants to signal that they no longer smell the previously delivered odorant are shown, i.e., the single choice (odorant no longer present) response delay between the opening of the washing-out channel and the key press given by the participant. Error bars signify +/- 1 standard error of the mean of the results of n = 22 participants.

Stimuli examples

Supplementary Figure 3 presents three different examples for each target fruit: a front-view image (left panels), a half-cut view (middle panels), and a lateral-view image (right panels). See *Methods* for detailed information.

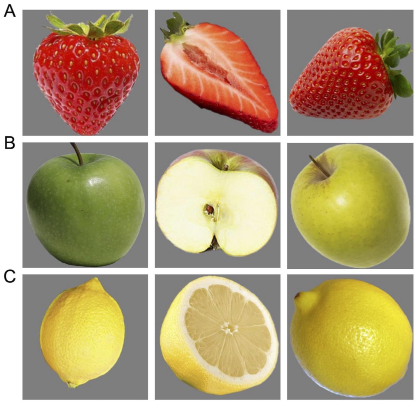

Supplementary Figure 3. Visual target fruits. (A) Strawberry. **(B)** Apple. **(C)** Lemon.

Supplementary Figure 4 shows fruits used as distractors in the tasks (one example for each).

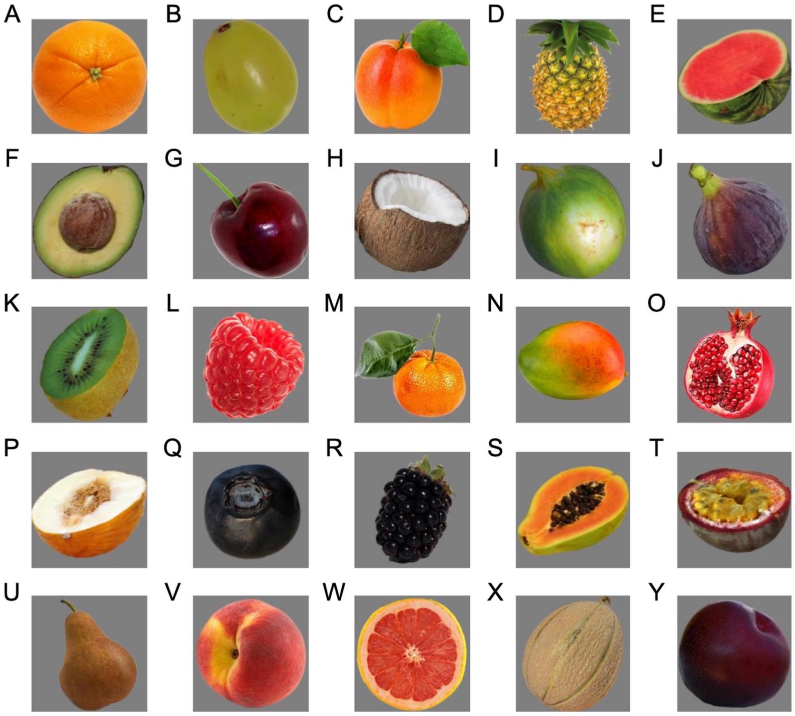

Supplementary Figure 4. Visual distractor fruits. (A) Orange. **(B)** Grape. **(C)** Apricot. **(D)** Pineapple. **(E)** Watermelon. **(F)**Avocado. **(G)** Cherry. **(H)** Coconut. **(I)** Barratiere melon. **(J)** Fig. **(K)** Kiwi. **(L)** Raspberry. **(M)** Mandarin. **(N)** Mango. **(O)**Pomegranate. **(P)** Melon. **(Q)** Blueberry. **(R)** Blackberry. **(S)** Papaya. **(T)** Passion fruit. **(U)** Pear. **(V)** Peach. **(W)** Grapefruit. **(X)**Muskmelon. **(Y)** Plum.

Effect of different odorants on visual search performance and response times

Supplementary Figure 5A shows that the visual-olfactory congruency effects on search performance did not vary across visual targets (strawberry, apple, lemon) or odorants (strawberry, apple, lemon). **Supplementary Figure 5B** shows the same pattern for response times. ANOVA reveals a significant main effect of congruency (performance: $F_{2,42} = 72.43$, $p < 0.001$; response time: $F_{2,42} = 97.87$, $p < 0.001$), but no significant effect of the type of fruit used as visual targets (performance: $F_{2,42} = 0.29$, *n.s.*; response time: $F_{2,42} = 0.08$, *n.s.*) or the type of fruit scent used as the odorant (performance: $F_{2,42} = 0.21$, *n.s.*; response time: $F_{2,42} = 0.4$, *n.s.*). The interaction term between congruency and visual target factors is not significant (performance: $F_{4,84} = 0.69$, *n.s.*; response time: $F_{4,84} = 0.38$, *n.s.*), as well between congruency and odorant factors (performance: $F_{4,84} = 0.98$, *n.s.*; response time: $F_{4,84} = 0.34$, n.s).

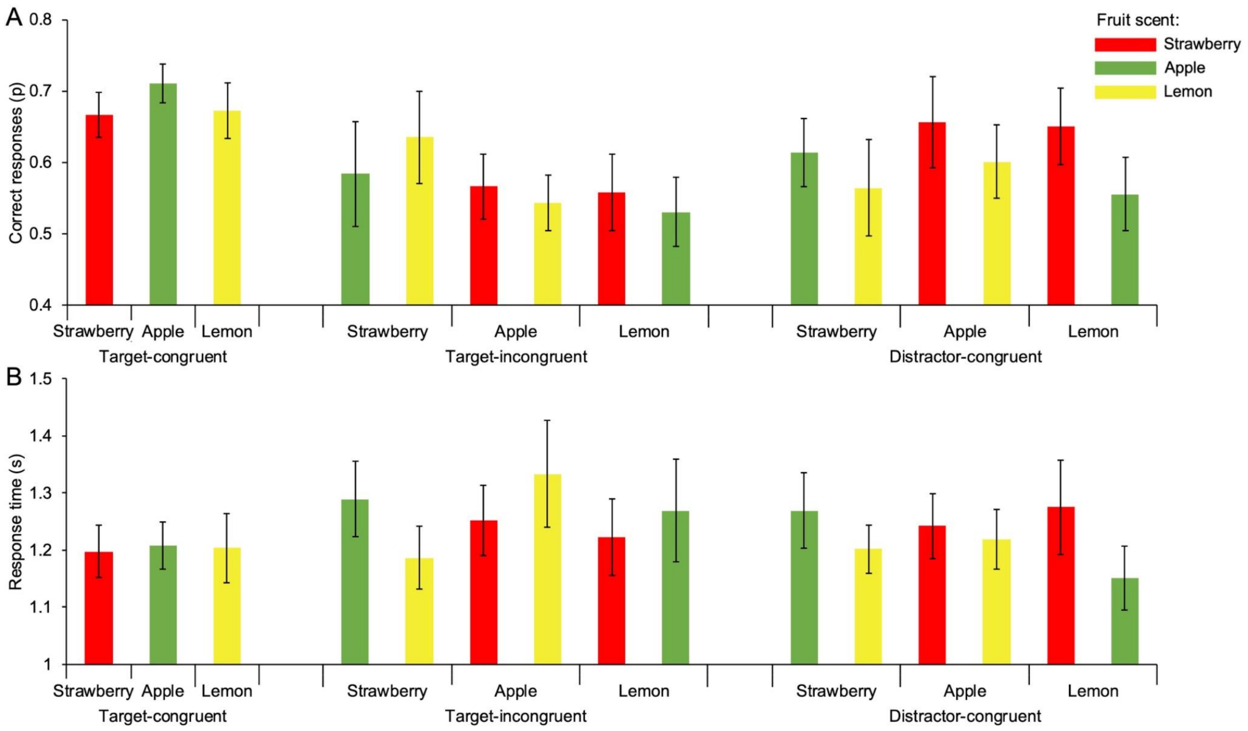

Supplementary Figure 5. Effect of different odorants on visual search performance. (A) Proportion of correct responses and **(B)** response times under different congruency conditions. The x-axis depicts the visual target, while the bar colors indicate the odorant. Error bars signify +/- 1 standard error of the mean.
